# Interplay between gut microbiota and acute kidney injury: a review of their mutual influence and causation

**DOI:** 10.1080/0886022X.2025.2522976

**Published:** 2025-07-01

**Authors:** Niroj Mali, Saroj Mali, Ling Zhang, Ping Fu

**Affiliations:** a Department of Nephrology and Kidney Research Institute, West China Hospital, Sichuan University, Chengdu, China; b School of Computer Science and Engineering, Central South University, Changsha, China

**Keywords:** Intestinal microbiota, acute kidney injury, short chain fatty acid, microbiome, gut microbiota, metabolome

## Abstract

It is unclear, therefore, exactly how gut microbes and renal processes communicate. Recent developments in the field of microbiome research present chances to enhance our comprehension and management of acute kidney injury (AKI). This review highlights important ideas and suggests more research while giving a general summary of the microbiome’s influence on kidney function and AKI. AKI is a complex condition that develops through a variety of pathways. Recent experimental research has highlighted the important role that the gut microbiota plays in the course of AKI. Our understanding of human biology has been completely transformed by the current increase in gut microbiota research. Amazing progress in DNA sequencing analysis, especially in the areas of metagenomics and metatranscriptomics, has greatly increased our ability to measure and track complex microbial populations. As such, we are now in a better position to look at how these communities affect human health and illness. As a result, the relationships between renal physiology and pathophysiology and the gut microbiota are now better understood. Both experimental AKI and chronic renal illness have been linked to changes in the intestinal microbiome. This succinct review attempts to examine our present knowledge of the immune-modulatory effects of the gut microbiota, how kidney disorders are influenced by it, and the possible reciprocal interaction between kidney diseases and the microbiota. Subsequent investigations ought to concentrate on delving deeper into the function of the gut microbiota in renal disorders and finding possible therapeutic strategies for adjusting it.

## 1. Introduction

### 1.1. A synopsis of the gut microbiome

Microbes are present in large quantities in the human body, most of which are found in the gut and plays vital role in human body. The gut microbiota is a complex and diverse colony of more than 100 trillion microbial cells. This community includes almost 1000 distinct species [1–3]. The Human Microbiome Project has demonstrated that millions of metabolites produced by the microbiome, which contains over 3 million genes compared to the 30,000 genes in the human genome, are important for human biology and the development of illness [4,5]. The majority of bacteria found in the gut are classified into four main phyla: *Actinobacteria*, *Firmicutes*, *Proteobacteria*, and *Bacteroidetes*. Over 90% of all bacteria belong to the phyla *Bacteroidetes* and *Firmicutes*, which are gut microbial species [6–10].

Recent studies have highlighted the substantial impact of the gut microbiota on human health, leading to increased interest in exploring its role in kidney diseases. Although a limited number of studies have investigated the gut microbiota in relation to kidney diseases, there is a growing body of research in this area [11].

The ‘hygiene hypothesis’ states that by inhibiting the normal development of immunological tolerance, a lack of early exposure to infectious agents, symbiotic microbes, and parasites increases vulnerability to immune-mediated illnesses [12,13]. Several variables, including oxidative stress, hemodynamic alterations, apoptosis, and inflammation, have a role in the development of AKI [14]. Since it recognizes that even a minor decline in kidney function, without total organ failure, can have important clinical consequences, the term ‘acute kidney injury’ (AKI) has replaced the term ‘acute renal failure’ (ARF). This acknowledgement emphasizes how serious renal disease may be and how it may affect the risk of complications and mortality [15]. In accordance with the Kidney Disease Improving Global outcomes Guidelines (KDIGO), AKI is classified into three stages, each of which is established by measuring the serum creatinine and urine output. Additionally, it is advised to assess patients for the possibility of developing AKI and to periodically check urine output and serum creatinine levels in both high-risk and AKI-affected persons [16]. The renal system and immune system are intimately related because the kidneys remove toxins from the body and keep an eye on blood-borne proteins, which helps to maintain the immune system’s equilibrium [17]. By filtering out circulating cytokines and bacterial toxins like lipopolysaccharide (LPS) and by continuously monitoring blood-borne proteins, the kidneys help maintain the integrity of the immune system. According to recent study, the development of intrinsic AKI may be influenced by diverse immune system components, renal tubular epithelial cells, and molecular variables [18].

Although the association between kidney illness and the gut microbiota has been extensively studied, the precise processes behind this interaction are still unknown. The gut microbiota plays a crucial role in AKI, and recent experimental and clinical research has highlighted the existence of a gut–kidney axis. The relationship between the gut microbiota and kidney tissue is known as the ‘gut–kidney axis,’ and research has shown that it is crucial to the onset and course of kidney damage [19,20]. An increasing amount of research has been conducted to support this idea, showing how important the gut microbiota is in kidney-related disorders [21–26]. Changes in the intestinal epithelial barrier’s integrity and an increase in the generation of toxins coming from the gut can result from disruptions in the gut microbiota. Renal damage may proceed more quickly as a result of these changes. Probiotics are living bacteria that, when given in large enough amounts, help the host’s health. They have been studied as a possible treatment for several chronic inflammatory disease models, including chronic kidney disease (CKD) [23].

## 2. Disorders associated with acute kidney injury and the gut microbial community

### 2.1. Kidney–gut crosstalk in AKI

**Figure 1.**
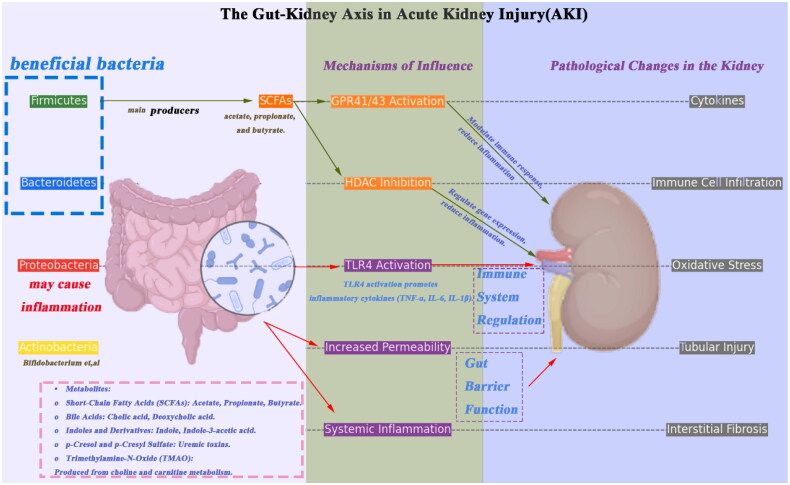
The gut–kidney axis in AKI. Caveat: Intra-phylum diversity: taxonomic classifications at the phylum level do not fully capture functional heterogeneity. For example: *Firmicutes* include both beneficial SCFA-producing genera (e.g., *Faecalibacterium*) and pathogens (e.g., *Clostridium difficile*). *Proteobacteria* encompass pro-inflammatory taxa (e.g., *Escherichia coli*) and beneficial species (e.g., *Helicobacter hepaticus*, which modulates hepatic immunity). Functional roles depend on strain-specific metabolic activity and host context.

Gut microbiota is a complex community of microorganisms that play a crucial role in human health and disease. The major phyla of gut microbiota include *Firmicutes*, *Bacteroidetes*, *Proteobacteria*, and *Actinobacteria*. These phyla produce various metabolites, such as short-chain fatty acids (SCFAs), bile acids, indoles and their derivatives, p-cresol (pC) and its sulfate, and trimethylamine-N-oxide (TMAO). These metabolites have significant effects on host metabolism and immune responses (Figure 1).

### 2.2. Pathophysiological framework

#### 2.2.1. AKI-induced gut dysfunction

Ischemia/reperfusion (I/R) injury → Intestinal hypoperfusion → Barrier disruption (‘leaky gut’).

Systemic inflammation → Microbial translocation.

#### 2.2.2. Dysbiosis-driven AKI aggravation

LPS/TLR4 activation → Tubular injury.

Impaired SCFA production → Loss of anti-inflammatory/antioxidant effects. Uremic toxins (e.g., *p*-cresol sulfate) → Oxidative stress.

#### 2.2.3. Microbiota dynamics across AKI trajectory pre-AKI state

Risk factors (CKD, diabetes, aging) associated with baseline dysbiosis.

Acute phase: Shifts in composition: ↓ diversity, ↑ Enterobacteriaceae, ↓ Lachnospiraceae*/*Ruminococcaceae.

Functional changes: ↓ butyrate production, ↑ urease/pC enzymes.

Recovery vs. non-recovery: persistent dysbiosis linked to fibrosis, CKD progression.

Sepsis-induced vs. non-septic AKI: contrasting microbiota patterns.

Gut microbiota influence kidney function through metabolites and the immune system, thereby affecting the pathological process of AKI. The left side lists the major components of gut microbiota and their metabolites, including SCFAs, bile acids, indoles and their derivatives, pC and its sulfate, and TMAO. The middle section describes how these metabolites modulate immune responses by activating G protein-coupled receptors (such as GPR41, GPR43) and Toll-like receptors (TLR4), leading to the production of inflammatory cytokines and systemic inflammatory responses through increased gut permeability. The right side shows pathological changes in the kidney during AKI, including inflammatory response, oxidative stress, and tubular injury. The bottom summarizes how gut dysbiosis leads to impaired gut barrier function, triggering systemic inflammation and exacerbating the severity of AKI. Modulating gut microbiota may help reduce the severity of AKI and improve kidney function.

### 2.3. Microbiome and uremic toxins

A microbiome is the collective genetic material of all the microbiota, which are groups of microorganisms living in a particular habitat. Since the development of 16S ribosomal RNA (rRNA) sequencing technologies, the importance of the microbiota to human health has been widely acknowledged. It contributes significantly to the explanation of the differences seen in the way different illnesses appear [2,25,27]. Previous research revealed that the gut microbiota of individuals with CKD produces uremic toxins, including trimethylamine N-oxide (TMAO), ammonia, and indoles [28–31]. Conversely, it is commonly believed that some metabolites, most notably SCFAs, are beneficial to health [30,32,33].

AKI outcomes are significantly influenced by the intestinal microbiome, according to recent experimental data. AKI pathophysiology has been linked to microbial SCFAs [21]. Acetate, propionate, or butyrate therapy was found to reduce kidney impairment in a mouse model of renal ischemia–reperfusion injury (IRI), as evidenced by a drop in plasma creatinine levels. Interestingly, treating mice with bacteria that make acetate had a similar effect. The authors hypothesize that histone deacetylases (HDACs) or the activation of SCFA GPCRs (G-protein coupled receptors) may be involved in the amelioration of IRI, potentially through the modification of epigenetic processes [32].

Chronic kidney disease is a highly prevalent and insidious disease, characterized by a gradual decline in renal function, particularly a reduction in filtration capacity. This decline leads to the accumulation of uremic toxins and affects many molecular mechanisms within the body. The relationship between CKD and dysbiosis is bidirectional: gut-derived metabolites and toxins influence the progression of CKD, while the uremic environment affects the microbiota. The accumulation of microbial metabolites and toxins is associated with increased risk of renal function loss and mortality. However, renal-protective metabolites such as SCFAs and bile acids can help restore renal function and improve survival rates in CKD patients. Specific dietary interventions that alter the gut microbiome can improve clinical outcomes in CKD patients. Low-protein and high-fiber diets increase the abundance of bacteria that produce SCFAs and anti-inflammatory bacteria. Fluctuations in the urinary microbiome are associated with increased susceptibility to infections and antibiotic resistance [34].

Dysbiosis of the gut microbiota plays a crucial role in the accumulation of protein-bound uremic toxins such as pC, indoxyl sulfate (IS), and p-cresyl sulfate (p-CS). These phenomena lead to increased levels of oxidative stress and inflammation, subsequently causing tissue damage and complications, particularly an increased cardiovascular risk, which is the leading cause of death in CKD. Additionally, CKD disrupts the colonic microbiome and its associated functions, affecting the gastrointestinal tract (GIT). This disruption leads to a loss of intestinal barrier integrity and an increased production of uremic toxins, resulting in impaired normal function of the GIT [35]. Studies have shown that genes such as GPX1, GSTO1, GSTO2, UMOD, and MGP are associated with CKD, further confirming the close relationship between renal pathology and gastrointestinal dysfunction [36]. IS, a microbial-derived uremic toxin, accumulates in late-stage CKD and causes renal and cardiovascular toxicity. IS is produced through the metabolism of indole, a compound generated exclusively by gut microbial tryptophanases. Research using (3S) ALG-05 treatment in mice demonstrated reduced cecal indole and serum IS levels with minimal changes in other tryptophan metabolic pathways [37]. This suggests that non-lethal gut microbial tryptophanase inhibitors could reduce IS in CKD.

### 2.4. SCFA and inflammation

#### 2.4.1. Microbiota-derived metabolites

Short-chain fatty acids, including butyrate, propionate, and acetate, are microbial metabolites whose availability in the gut and other organs depends on environmental factors such as diet and antibiotic use, which determine the diversity and metabolism of the microbiota. SCFAs regulate epithelial barrier function and mucosal and systemic immunity through evolutionarily conserved processes involving G-protein-coupled receptor signaling or HDAC activity [38]. SCFAs are capable of modulating the polarization of T-cell subsets, promoting the generation of regulatory T cells (Tregs), thereby suppressing inflammatory responses. SCFAs can also inhibit the function of neutrophils, reducing the production of pro-inflammatory mediators. In clinical studies, the levels of certain metabolites in the blood (such as TMAO) are associated with the risk of progression from AKI to CKD. Antibiotic-induced depletion of the gut microbiota can alleviate inflammation and oxidative stress during the transition from AKI to CKD [39].

AKI may be influenced by the gut microbiota, according to recent research. The gut microbiota produces SCFAs, which have anti-inflammatory qualities and are thought to have renoprotective benefits [32]. Four SCFA receptors have been identified as being expressed in the kidney: G-protein-coupled receptors: GPR41, GPR43, Olfr78, and GPR109a. GPR41 and GPR43 expression has been found in both the renal artery and the whole kidney using reverse-transcription polymerase chain reaction (RT-PCR) [40]. Treatment with the three primary SCFAs – acetate, propionate, and butyrate – has been demonstrated in animal models to ameliorate injury-induced renal impairment. Reactive oxygen species, inflammation, immune cell infiltration, and apoptotic cells in the injured kidneys are all decreased in conjunction with this improvement. Furthermore, SCFAs influence the DNA methylation state and encourage kidney epithelial cell proliferation. The hygiene hypothesis is another potential way by which the microbiota influences AKI outcomes; however, more investigation is required to completely comprehend these mechanisms [41].

GPR43 expression was upregulated in kidney tissue when acetate was administered in a mouse model of IRI [32,42]. Conversely, Olfr78, which mediates renin secretion in response to SCFAs, is expressed in the renal juxtaglomerular apparatus [40]. It has been demonstrated that SCFAs are useful in cases of AKI. By regulating inflammation, apoptosis, and autophagy, SCFA therapy decreased kidney damage in a mouse model of acute renal injury. Reactive oxygen species and inflammatory cytokines were reduced by SCFAs, whereas Toll-like receptor 4 expression and nuclear factor kappa-light-chain enhancer (NF-κB) activation were hindered. Additionally, dendritic cell maturation and T-cell proliferation were restrained. Additionally, SCFAs enhanced mitochondrial activity and shielded kidney epithelial cells from harm brought on by hypoxia. According to these results, SCFAs may be used as therapeutic agents for AKI since they target a variety of pathways related to inflammation and damage [32]. In a rat model of contrast-induced nephropathy, sodium butyrate treatment lowered inflammation and IL6 levels, blocking NF-κB translocation and indicating the possibility of modulating inflammation and protecting AKI [43]. Although earlier research has shown that GPR109a is present in the adipose tissue of the kidney, its precise function in kidney disease is still unknown [44]. It has been demonstrated that GPR109a stimulates the development of T cells that generate interleukin-10 and Tregs [45]. Furthermore, it triggers apoptosis and suppresses NF-κB activity by methods unrelated to the inhibition of HDAC. These are believed to be associated with butyrate’s anti-inflammatory and tumor-suppressive properties [46].

SCFAs, such as propionate and butyrate, can alleviate renal inflammation and fibrosis through multiple mechanisms. For example, SCFAs inhibit the production of pro-inflammatory cytokines and reduce renal inflammation by binding to G-protein-coupled receptors (such as GPR41 and GPR43) [47].

As the disease progresses, the levels of propionate and butyrate in the feces of CKD patients gradually decrease and are closely related to established clinical parameters such as serum creatinine, blood urea nitrogen, and estimated glomerular filtration rate. *In vivo* studies have shown that administration of propionate and butyrate before or shortly after injury can prevent and slow the progression of damage. This is manifested by reduced renal injury markers, decreased expression of pro-inflammatory and fibrotic markers, and long-term recovery of renal function [48].

Multiple studies have found that various traditional Chinese medicine polysaccharides can modulate the gut microbiota, especially probiotics, to produce beneficial effects on human health. For example, after treatment with *Astragalus polysaccharide (APS)*, the abundance of SCFA-producing bacteria (such as *Kineothrix*, *Faecalibaculum*, *Akkermansia*, *Lactobacillus*, and *Roseburia*) is upregulated. In APS-supplemented mice with adenine-induced kidney injury, levels of acetate, propionate, and butyrate were increased. APS supplementation significantly increased the levels of GPR41 and GPR43 in the kidneys of adenine-induced mice and partially increased the level of GPR109a. This study confirmed that APS supplementation can upregulate the abundance of SCFA-producing bacteria and increase SCFA levels, thereby alleviating tubulointerstitial injury and fibrosis through GPRs [49].

In addition, SCFAs regulate immune cell functions through multiple mechanisms to reduce inflammation, mainly by inhibiting the production of pro-inflammatory cytokines (such as TNF-α and IL-6) and promoting the differentiation of Tregs. A study induced experimental anti-glomerular basement membrane (GBM) disease in Wistar Kyoto rats by immunizing them with a renal T-cell epitope α3127-148 and intervened with 150 mM sodium acetate, propionate, or butyrate in drinking water from day 0 to day 42. The results showed that SCFA treatment (especially butyrate) alleviated anti-GBM nephritis in the rat model, indicating its potential therapeutic application [50]. SCFAs can also reduce systemic inflammation by modulating gut barrier function and decreasing the production of inflammatory mediators. In an asthma animal model, both butyrate and propionate inhibited the M2 polarization pathway, thereby reducing allergic airway inflammation. This may be closely related to butyrate’s activation of the GPR43 receptor and inhibition of the NF-κB signaling pathway, reducing inflammatory cell infiltration [51].

This indicates that SCFAs produced by the fermentation of carbohydrates by gut microbiota play a crucial role in regulating host physiological functions. Among them, acetate, propionate, and butyrate are key participants in various biological processes. Recent studies have revealed their important functions in immune and inflammatory responses.

#### 2.4.2. The contribution of the gut microbiota

According to a study by Jang et al. when germ-free (GF) animals were subjected to ischemia-induced AKI, they showed greater impairments to their renal function, inflammation, and damage than control mice [41]. This might be explained by an immunological response of the T-helper 1 type, which is comparable to what is seen in autoimmune illnesses. Furthermore, by exhibiting immunomodulatory effects, specifically through altering the polarization of T-cell subsets and natural killer cells, the microbiome may have a wider impact on autoimmune kidney disorders [52,53]. In another study, broad-spectrum antibiotics were given to mice to protect them against kidney damage brought on by ischemia–reperfusion. A reduction in the maturation of bone marrow-derived monocytes and F4/80+ renal-resident macrophages was associated with this protection. Notably, the protective effect was eliminated when fecal material from animals without treatment was transferred into mice whose microbiota had been compromised. These findings suggest that the gut microbiota is important in mediating the protective effects of broad-spectrum antibiotics against ischemia–reperfusion-induced kidney damage [54]. In another study, we found that the gut microbiome protects kidney health. In comparison to normal mice, we discovered that kidney damage caused by I/R was more severe in GF animals. Nevertheless, when transplanted normal mouse feces into GF mice, the severity of this aggravated harm was reduced. This implies that in the setting of I/R, the intestinal microbiome may have a function in preventing renal damage [24].

According to Yang et al. pretreatment with *Bifidobacterium bifidum BGN4* decreased renal IRI severity, attenuated dysbiosis and gut barrier disruption caused by AKI, and expressed less interleukin-17A. This suggests that BGN4-induced immunomodulation plays a role in its renoprotective effect [55]. Previous studies have demonstrated a reciprocal interaction between the kidney and the gut in cases of AKI. They discovered that intestinal dysbiosis, a disorder caused by abnormalities in the microbial makeup of the gut, was brought on by IRI. The results of the kidney after ischemia damage were significantly influenced by this altered intestinal microbiome. The immune system was significantly impacted by the dysbiotic microbiota, which in turn affected the response and eventual results of the post-ischemic kidney. These results emphasize the relationship between the kidney and the intestine in AKI and stress the role the gut microbiota plays in influencing the immune system and general renal function in this illness [56].

Higher blood levels of IS were associated with a higher death rate in hospital-acquired AKI patients, according to a prospective cohort research [57]. Furthermore, *Lactobacillus salivarius*’s suppression of indole and p-cresyl synthesis in a mouse model showed protective benefits against cisplatin-induced kidney damage [58].

### 2.5. Hemodynamics

Vasoconstriction and vasodilation of blood vessels play a crucial role in regulating blood pressure [59]. Research has shown a link between gut microbiota and hypertension (HTN). For instance, studies have observed changes in the gut microbiome, referred to as ‘dysbiosis,’ in hypertensive individuals, including findings from mice, rats, and humans [59–62]. AKI is frequently accompanied by HTN. Angiotensin II injection was found to raise blood pressure in normal mice with a normal gut microbiota, but not in GF mice, who do not have a gut microbiota [63]. Genes involved in vascular monocyte chemoattractant protein 1 (MCP-1), inducible nitric oxide synthase (iNOS), and NADPH oxidase component (Nox2) were expressed less in GF mice treated with angiotensin II than in normal mice. Furthermore, the important transcription factor linked to the manufacture of IL-17, retinoic-acid receptor-related orphan receptor gamma t (Rorγt), was shown to be downregulated [63]. These results led to a decrease in vascular leukocyte adherence as well as a decrease in Ly6G(+) neutrophil and Ly6C(+) monocyte infiltration into the aortic artery wall. This implies that the immune cell infiltration and subsequent vascular inflammation caused by the gut microbiota may be involved in the HTN brought on by angiotensin II treatment. Moreover, a different study showed that mice given a diet rich in salt (2% NaCl in drinking water) had HTN and kidney impairment. The transplantation of these mice’s feces into normal mice caused kidney damage linked to HTN and a ‘leaky gut’ syndrome [64]. Salt intake causes gut microbiota imbalance, leading to renal damage. SCFAs produced by gut microbiota may regulate blood pressure, suggesting a potential association between gut microbiota and blood pressure regulation.

In patients with AKI who experience hypotension, the microbiota exerts various effects. The occurrence of AKI is often accompanied by changes in the composition and structure of the microbiota, that is, dysbiosis. Hypotension, as an additional stress factor, can further disrupt the balance of the microbiota. For example, the proportion of beneficial bacteria such as *Bifidobacterium* and *Lactobacillus* may decrease, while the abundance of some harmful bacteria may increase. At the same time, SCFAs have the ability to induce vasodilation [65,66]. This process can subsequently lead to a reduction in HTN [67,68]. The vasodilatory effects of acetate, propionate, and butyrate contribute to their ability to lower blood pressure. When administered acutely, such as through intravenous or intraperitoneal methods, SCFAs can induce rapid hypotension that occurs within seconds and typically resolves within minutes [40,69]. Additionally, long-term consumption of acetate, butyrate, or propionate also leads to reduced blood pressure [70–73]. Notably, SCFAs can lower blood pressure even in the absence of prebiotic dietary sources [74].

### 2.6. Septic AKI

About 45–70% of cases of AKI are linked to sepsis, making it a common risk factor for the development of AKI [75]. Research has indicated that individuals with sepsis-associated acute kidney injury (SA-AKI) exhibit greater rates of renal damage and a higher severity of injury than those without SA-AKI [76]. Another study found that in patients with septic shock, the incidence of SA-AKI ranged from 11 to 31% and as high as 41–78% [77]. Anaerobic bacteria, in particular, are lost when septic AKI occurs, altering the gut microbiota’s makeup. Antibiotic therapy, parenteral nutrition, systemic inflammation, and ‘leaky gut’ syndrome are among the variables that might lead to dysbiosis of the gut microbiota. Additionally, the use of parenteral nutrition and decreasing gut microbiota abundance lead to decreased synthesis of SCFAs, which have immune-modulating qualities [78]. Based on the earlier mentioned ways in which the gut and kidneys interact during sepsis, several studies have suggested that addressing SA-AKI should involve targeting the gut microbiota. SA-AKI can be classified into three subgroups based on its underlying mechanism. To address disorders related to the gut microbiota, three approaches have been proposed: (i) the use of probiotic supplements, (ii) selective digestive tract decontamination (SDD), and (iii) microbial restoration therapy [23,79,80].

The findings of this study suggest that the involvement of microbiota in experimental AKI is complex, as do the findings of other direct studies on the subject. It emphasizes the necessity of carefully planned follow-up studies to determine the exact pathophysiological function of the gut microbiota in AKI. Note that there was no published original study on the impact of AKI on the gut microbiome or vice versa at the time this publication was written when searches for phrases like ‘human AKI and microbiome’ or ‘human AKI and bacteria’ were conducted on PubMed.

Table 1 focuses on the experimental models of AKI and the methods used to assess changes in gut microbiota and delves into the experimental frameworks employed to study AKI and the methodologies utilized to analyze alterations in gut microbiota. Understanding these models and analytical techniques is crucial for elucidating the interplay between kidney injury and gut health. (1) Experimental models of AKI various models are used to induce AKI in research settings, each offering unique insights into the pathophysiology of the condition: IRI: this model simulates kidney injury caused by a temporary loss of blood flow followed by restoration. It mirrors clinical scenarios such as acute tubular necrosis, allowing researchers to investigate how gut microbiota respond to ischemic conditions. *Nephrotoxic models*: These involve the administration of nephrotoxic agents, such as cisplatin or gentamicin, to induce AKI. These models help explore the mechanisms of toxicity and the subsequent effects on gut microbiota composition. *Sepsis-induced AKI*: Models that simulate sepsis, a common cause of AKI, are crucial for understanding systemic inflammation’s role in kidney injury and how this alters gut microbiota dynamics. *Obstructive models*: These models mimic kidney obstruction (e.g., ureteral obstruction), providing insights into how prolonged pressure and reduced perfusion affect both renal and gut health. (2) Assessing changes in gut microbiota analyzing gut microbiota in the context of AKI involves several methodologies: 16S rRNA gene sequencing: this technique allows for the comprehensive profiling of bacterial communities in fecal samples. By sequencing the 16S rRNA gene, researchers can identify and quantify various microbial taxa, revealing shifts in composition associated with AKI. *Metagenomic sequencing*: Unlike 16S rRNA sequencing, metagenomic sequencing provides a broader view by analyzing all genetic material present in a sample. This approach can uncover functional potential and metabolic pathways of the gut microbiota that may influence kidney function. *Metabolomics*: This method examines the metabolites produced by gut bacteria, such as SCFAs and uremic toxins. Understanding these metabolites can elucidate their roles in kidney health and disease. *Culture techniques*: Although less comprehensive, traditional culture methods can isolate specific bacterial strains from gut samples. This approach is useful for studying the properties and effects of particular microbes on kidney function. (3) Integrative approaches combining these models and analytical methods allows researchers to explore the gut–kidney axis more effectively. For instance, correlating changes in gut microbiota with kidney injury markers can provide insights into how gut health influences renal outcomes. (4) Clinical relevance findings from these experimental models can inform clinical strategies aimed at gut microbiota modulation in AKI patients. Understanding the microbiota’s role in kidney injury may lead to innovative therapeutic approaches, such as probiotics or dietary interventions, to support kidney health. The exploration of AKI models and gut microbiota analysis is pivotal for advancing our understanding of the interactions between kidney injury and gut health. By employing various experimental techniques, researchers can uncover mechanisms that may ultimately inform new therapeutic strategies for managing AKI and improving patient outcomes.

**Table 1.** AKI models: implications for gut microbiota studies.

Author	Population	Parameter assessed	Outcome	Results
Jang et al. [41]	Germ-free mice that are then fed a diet high in bacteria	T cell and NK cell counts and morphologies, and cytokine panel	Degree of renal damage and reduction in function following IRI	Microbiological stimuli affect renal lymphocyte phenotypes and lessen the severity of renal damage.
Long et al. [81]	C57BL/6 mice	Influence of elevated Hcy levels	Cisplatin-induced AKI	Cisplatin induces more severe tubular injury, tubular cell apoptosis, and lower proliferation in hyperHcy mice
Li et al. [82]	C57BL/6 mice and germ-free C57BL/6 mice	Microbiota composition	Severity of IRI	Intestinal dysbiosis, inflammation, and leaky gut are consequences of AKI but also determine its severity
Mishima et al. [83]	Germ-free mice and mice with microbiota	Metabolome analysis	Extent of kidney damage in adenine-induced AKI	Germ-free mice enhanced host purine metabolism and exacerbated kidney damage
Machado et al. [43]	Wistar rats	SCFA (sodium butyrate)	Creatinine levels, inflammatory markers, and histological alterations were evaluated in relation to AKI caused by contrast.	SCFA treatment attenuated creatinine levels and histological damage
Sun et al. [84]	Sprague-Dawley rats	SCFA (sodium butyrate)	Levels of creatinine, AKI markers, antioxidant enzymes, and histology in gentamicin-induced AKI	Chronic treatment with SCFA protects from gentamicin-induced nephrotoxicity
Wang et al. [57]	Prospective observational design: 262 patients with hospital-acquired AKI	Serum indoxyl sulfate levels	90-Day mortality	Serum indoxyl sulfate levels were higher in patients with acute kidney injury (AKI), and this was associated with a worse prognosis.
Veldeman et al. [58]	Prospective observational design: 194 patients with sepsis	Serum indoxyl sulfate and p-cresyl sulfate levels	Acute kidney injury due to sepsis	Serum indoxyl sulfate and p-cresyl sulfate levels were higher in patients with AKI and correlated with AKI course
Andrade-Oliveira et al. [32]	C57BL/6 mice	SCFAs (acetate, butyrate, propionate)	Levels of creatinine and urea, necrosis score in kidney tubular epithelial cells in IRI	Mice treated with acetate-producing bacteria had improved mitochondrial biogenesis and better outcomes
Al-Harbi et al. [42]	BALB/c mice	SCFA (sodium acetate)	Kidney function/renal peroxidase activity/kidney tubular structure in sepsis-induced AKI	Acetate ameliorates sepsis-induced kidney injury by restoration of oxidant–antioxidant balance in T cells
Lee et al. [85]	Sprague-Dawley rats and Caco-2 cells	*Lactobacillus salivarius* BP121	Cisplatin-induced AKI occurrence	The administration of *L. salivarius* BP121 reduced Caco-2 cell damage and offered defense against M. cisplatin-induced AKI.
Zheng et al. [86]	BALB/c mice and Bama miniature pigs	Microbial cocktail (*Escherichia*, *Bacillus*, *Enterobacter*)	In nephrotoxin-induced acute kidney damage (AKI) brought on by glycerol, cisplatin, and adenine, the levels of urea and creatinine were changed.	In both murine and porcine models of AKI, the orally delivered cocktail reduced urea and creatinine concentration
Dong et al. [87]	Retrospective analysis, 176 cirrhotic adult patients (88 treated with rifaximin	Rifaximin	AKI and HRS risk	AKI, hepatorenal syndrome (HRS), and renal replacement treatment (RRT) incidence rate ratios were all lower in the rifaximin group.
Nakade et al. [24]	C57BL/6 mice I/R unilateral 40 min	16S rRNA gene-sequencing analysis	Increased *Lactobacillus*, *Clostridium*, *Ruminococcus* Decreased *Bifidobacterium* TM7	Increased d-serine/l-serine
Yang et al. [56]	C57BL/6 mice I/R bilateral 25.5 min (SPF mice) 28.5 min (GF mice)	16S rRNA gene-sequencing analysis	Increased *Enterobacteriaceae* Decreased *Lactobacilli Ruminococcaceae*	Decreased SCFA
Andrianova et al. [26]	Wistar rats I/R unilateral 40 min	Metagenomic analysis	Increased *Staphylococcus, Prevotella*	Increased 32 acylcarnitines Decreased tyrosine, tryptophan, proline
Zhu et al. [23]	Individuals with CKD in stages 3–5 (*n* = 62) were enrolled and underwent randomization to receive either a placebo (*n* = 29)	Clinical lab work	Creatinine, BUN, parathyroid hormone (PTH), and the estimated glomerular filtration rate (eGFR)	In the probiotic group, serum cystatin C level decreased
Zhu et al. [23]	Bilateral renal ischemia–reperfusion (I/R)-induced gut microbial dysbiosis	16S rRNA gene sequencing analysis	The population of *Bacteroidetes* was more abundant in the group that had pretreated with Lac.z.	Administration of *L. casei* Zhang reduced renal damage and delayed the development of CKD in rats. Fatty acids SCFAs and nicotinamide levels in the serum and kidney increased after the *L. casei* Zhang treatment, which reduced kidney inflammation and protected renal tubular epithelial cells from harm.
Osada et al. [88]	Increased tubular injury Lower glucose and pyruvate levels in antibiotic-treated mice (vs. control) after IRI	Reduced SCFA and 16S rRNA quantity in antibiotic-treated mice	Decreased pyruvate levels in the kidney, which may have been caused by the activation of renal gluconeogenesis	Increase the vulnerability of the kidney to ischemia–reperfusion injury
Hsiao et al. [89]	CP plus *C. butyricum* and *L. reuteri* (10 days after CP) vs. CP only control in Wistar rats	Lower inflammation (KIM-1, F4/80, MPO), fibrosis (collagen IV, fibronectin, a-SMA), blood endotoxin, and indoxyl sulfate	Increase in *Bifidobacterium*, *Ruminococcaceae*, *Ruminiclostridium_9*, and *Oscillibacter*. Decrease in *Escherichia Shigella*	
Chavez-Iniguez et al. [90]	A total of 92 patients with AKI associated with sepsis were randomized, 48 to the probiotic and 44 to placebo group	Evaluate the effect on KFR, mortality, kidney replacement therapy (KRT), urea, urine volume, serum electrolytes, and adverse events at day 7	Significant reduction in urea levels from 154 to 80 mg/dl (*p* = .04), though no mortality benefit	Probiotics for 7 days did not improve KFR or clinical outcomes, but were safe

### 2.7. Gut microbiota-immune system interaction in kidney disease

The intricate relationships between humans and bacteria have been shaped by their millions of years of coevolution. Targeted immune responses to various bacteria are made possible by the mutually beneficial association that has developed between the gut microbiota and vertebrates as a result of the evolution of adaptive immunity. However, dysbiosis, which results from a disturbance of this evolutionary equilibrium, can aid in the emergence of illness [91].

Gut microbiota plays a crucial role in modulating both local and systemic inflammatory profiles through direct and indirect interactions with the host. Probiotic and commensal bacteria strengthen the gut barrier function by increasing the secretion of antimicrobial peptides (e.g., β-defensin), enhancing mucus production, stabilizing tight junctions between intestinal epithelial cells, and suppressing inflammatory responses [92]. These mechanisms prevent the leakage of bacterial products such as LPS into the circulation, thereby reducing systemic inflammation [93].

Conversely, dysbiosis is characterized by a reduction in beneficial bacteria and an increase in pro-inflammatory phyla such as *Proteobacteria* and *Actinobacteria*. This imbalance leads to increased intestinal permeability and a shift in the Th17/Treg balance, favoring pro-inflammatory Th17 cells over anti-inflammatory Tregs. Such changes can exacerbate systemic inflammation and contribute to the development of chronic diseases, including kidney disease [94].

Targeting the gut microbiota through dietary interventions, probiotics, or microbiome-based therapies holds promise for mitigating inflammation and improving outcomes in CKD. For example, interventions that promote the growth of beneficial bacteria (e.g., *Lactobacillus*, *Bifidobacterium*) and enhance the production of anti-inflammatory metabolites (e.g., SCFAs) may help restore gut homeostasis and reduce systemic inflammation [95,96]. Future research should focus on elucidating the mechanisms underlying gut–kidney interactions and developing personalized strategies to optimize gut microbiota composition in CKD patients.

### 2.8. Gut microbiota influence immune modulation

*SCFA – immune cell crosstalk*: SCFAs (e.g., butyrate, acetate, and propionate) modulate immune responses through GPCRs such as GPR43 and GPR109A. These receptors are expressed on neutrophils, dendritic cells, and T cells, where their activation suppresses pro-inflammatory cytokines (e.g., IL-6, TNF-α) and promotes the differentiation of anti-inflammatory Tregs [45,46].

*TLR – microbial ligand interactions*: Gut microbiota interacts with host immune cells through pattern recognition receptors such as TLR4. For instance, LPS from Gram-negative bacteria can trigger TLR4 signaling, leading to NF-κB activation and a cascade of pro-inflammatory cytokines that exacerbate renal injury in AKI [54].

*Th17/Treg imbalance in dysbiosis*: Dysbiosis leads to a shift in the gut mucosal immune landscape toward a Th17-dominant response, enhancing IL-17-mediated inflammation and weakening the anti-inflammatory Treg pathway. This shift has been directly linked to worsened outcomes in ischemia–reperfusion and sepsis-induced AKI models [55].

*Gut barrier and systemic inflammation*: Loss of epithelial barrier integrity increases translocation of microbial products (e.g., LPS, peptidoglycans) into systemic circulation, amplifying systemic inflammation through innate immune activation. This ‘leaky gut’ phenomenon plays a central role in immune-mediated kidney injury [29].

#### 2.8.1. Mechanisms of microbiota-kidney crosstalk

*Immunomodulation*: Treg/Th17 imbalance driven by SCFAs. Neutrophil activation via gut-derived DAMPs (damage-associated molecular patterns).

*Metabolite signaling*: SCFAs (butyrate) → HDAC inhibition → Nrf2 activation (nuclear factor erythroid 2-related factor 2) → renal protection.

TMAO → NLRP3 (NLR family pyrin domain containing 3) inflammasome → tubular damage.

*Barrier integrity*: Butyrate → tight junction enhancement → reduced endotoxemia.

## 3. Therapeutic targets

**Figure 2.**
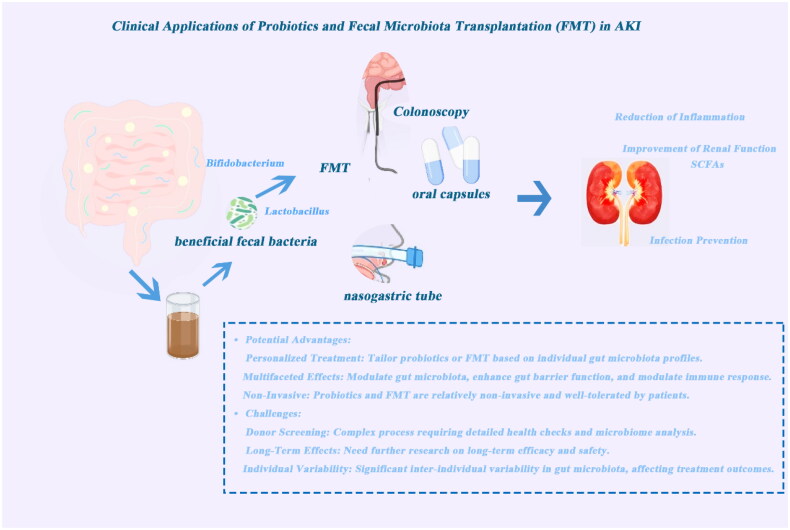
Clinical applications and mechanisms of probiotics and fecal microbiota transplantation (FMT) in acute kidney injury (AKI).

The mechanisms of probiotics, include modulation of the gut microbiota, enhancement of intestinal barrier function, and immune regulation. The central section details the process of fecal microbiota transplantation (FMT), from donor selection and fecal processing to transplantation methods. Clinical applications of probiotics and FMT in AKI, such as reducing inflammation, improving renal function, and preventing infection. The bottom section highlights potential advantages and challenges, including personalized treatment, multifaceted mechanisms, noninvasive nature, and issues related to donor screening, long-term efficacy, and individual variability (Figure 2).

### 3.1. Examining upcoming studies and possible therapeutic uses of microorganisms in renal diseases

Recent studies have shown that gut microbiota-derived d-serine can protect against AKI by reducing inflammation and promoting renal recovery [90]. Additionally, probiotic administration has been demonstrated to reduce mortality and improve intestinal epithelial homeostasis in experimental models of sepsis [97]. These findings suggest that modulating the intestinal microbiome could be a promising therapeutic strategy for AKI. A retrospective analysis of individuals with cirrhosis who were treated with rifaximin showed a lower incidence of AKI. The observed protection was attributed to modifications in the gut microbiota as a consequence of rifaximin therapy, instead of a total eradication of gut microflora. However, this study did not include any microbiota-related assessments [87]. Furthermore, a metagenomic study demonstrated that maintenance of immunosuppressive medication affected the fecal microbiome of kidney transplant patients. In a similar vein, changes in the fecal microbiota were noted in adult liver transplant recipients. Changes in the gut microbiota have also been seen in studies examining the progression from AKI to CKD and end-stage renal disease (ESRD). Notably, after uremia, recent studies have revealed changes in the intestinal flora of ESRD patients.

Research is needed to better understand these pathways and their possible ramifications, as the field of examining microbial signaling pathways in relation to kidney illnesses is still in its early stages. However, it is interesting to think about the changes that might present themselves in the future for applying microbial signaling pathways for therapeutic applications. First off, examining the relationship between dysbiosis and illness may offer insightful and surprising information about the fundamental processes of pathophysiology. Second, investigating whether dysbiosis causes or maintains disease may lead to novel therapeutic approaches that may be advantageous to patients.

Research on the function of microbiota in renal diseases is still in its infancy, even though its significance in many human diseases is being actively studied. The majority of kidney disease treatments now available concentrate on providing supportive care, underscoring the necessity for innovative strategies to improve outcomes in these disorders. Research investigations into the involvement of microbiota in renal illnesses hold great promise for improving our comprehension of the underlying processes and opening up new avenues for the development of therapeutic therapies.

The relationship between the kidney and the gut during AKI may be interpreted as follows, based on the previously cited studies:

1. AKI causes disruptions in the gut microbiota, which lead to dysbiosis of the intestinal tract and breaching of the intestinal barrier – a condition often known as ‘leaky gut.’
2. The immune responses inside the gut mucosa are altered by dysbiosis and leaky gut. As a result, pro-inflammatory macrophages and neutrophils gather, and the Th17 pathway is activated, further fostering inflammation.
3. Through systemic inflammation, the change in mucosal immunity toward a proinflammatory state might worsen kidney damage and impact other organs.
4. By reestablishing a balanced gut microbiota, probiotics, or their metabolites may protect the kidneys.

In conclusion, dysbiosis, disruption of the gut barrier, immunological responses, and systemic inflammation all interact with one another during the crosstalk between the gut and kidney during AKI. Renoprotective benefits may be provided by probiotics or their metabolites through immune system modulation, gut health improvement, and dysbiosis correction.

### 3.2. Probiotics for kidney disease

*Lactobacillus casei* Zhang, a probiotic included in traditional Chinese koumiss, may protect mice against AKI and CKD, according to recent research. Furthermore, it was noted that the probiotic could be able to halt the advancement of CKD in people. The modification of the gut microbiota and increased availability of nicotinamide and SCFAs that reduce inflammation are the mechanisms by which these benefits are mediated [23]. In the same study [23], it was found that administering *L. casei* Zhang to patients with CKD decreases their kidneys’ deterioration. Participants with stage 3–5 CKD (*n* = 62) were recruited and randomly assigned to receive either a placebo (*n* = 29) or probiotic (*n* = 1). Serum cystatin C (a measure of renal function) increased significantly in the placebo group from 3.00 ± 0.18 to 3.18 ± 0.22 mg/L (*p* = .017), while the *L. casei* Zhang supplement resulted in a significantly lower level of cystatin C compared with the placebo group (*p* = .007). Additionally, after three months, serum PTH increased considerably (*p* = .036) in the placebo group but decreased in the probiotic group throughout the same period. The fascinating results of Zhu et al. will motivate more investigation into the practical use of *L. casei* Zhang’s possible health-promoting benefits in renal illness. Preclinical data suggest that supplementing with *L. casei* Zhang before a programmed intervention known to cause a high incidence of AKI, such as cardiovascular surgery, cisplatin chemotherapy for cancer, or patients who are at high risk of AKI upon hospital admission, is the most likely scenario for clinical validation [98,99].

In contrast to CKD, as demonstrated in this study, this type of clinical trial with probiotics long-term impact on AKI may be conducted to see actual benefits in the future.

### 3.3. Potential adverse events of probiotics in AKI

The use of probiotics in patients with AKI has been explored for their potential benefits in modulating gut microbiota and improving kidney function. However, there are also potential adverse events associated with their use. Here are some key points regarding the adverse events of probiotics in the context of AKI: common adverse events: the most frequently reported adverse events related to probiotics include gastrointestinal symptoms such as abdominal distension, nausea, vomiting, and diarrhea. These events were monitored in clinical trials and were found to be similar between probiotic and placebo groups, indicating that probiotics may be safe for use in this population [100].

*Lack of significant improvement*: In a clinical trial involving patients with sepsis-induced AKI, the administration of probiotics did not lead to significant improvements in kidney function recovery or mortality rates. While some biochemical markers like urea levels showed improvement, the overall safety profile was maintained without an increase in adverse events compared to placebo [100]. *Immunomodulatory effects*: Probiotics may exert immunomodulatory effects, which could theoretically lead to adverse outcomes in certain populations, particularly those with compromised immune systems. The modulation of immune responses can sometimes result in unintended consequences, such as increased susceptibility to infections or inflammatory responses [55].

*Potential for dysbiosis*: Although probiotics are intended to restore healthy gut microbiota, there is a possibility that they could contribute to dysbiosis if not appropriately selected or administered. This dysbiosis could exacerbate kidney injury or lead to other systemic complications [90].

*Specific populations at risk*: Patients with severe comorbidities or those undergoing immunosuppressive therapies may be at higher risk for adverse events when using probiotics. The interaction between probiotics and the underlying health conditions of these patients needs careful consideration [90]. While probiotics may offer benefits in managing AKI through gut microbiota modulation, their use is not without risks. Adverse events primarily involve gastrointestinal symptoms, and careful monitoring is essential, especially in vulnerable populations.

### 3.4. Microbial replacement therapies

#### 3.4.1. Fecal microbiota transplantation and SCFAs

FMT is a microbial replacement therapy that restores gut microbiota balance, promoting anti-inflammatory, antioxidant, and beneficial metabolite production by intestinal probiotics [101]. FMT was reported by Assimakopoulos et al. to have a positive effect on intestinal barrier integrity. They found that FMT enhanced the expression of proteins involved in tight junctions, such as occludin (by 56 ± 15%) and claudin-1 (by 84 ± 7%), and prevented the activation of NF-κB. Furthermore, by augmenting the paneth cell population and diminishing the production of inflammatory molecules, FMT was able to reestablish the regulation of immune activity [102]. In another study, it was demonstrated that FMT can restore the gut microbiota by promoting the growth of beneficial bacteria and increasing the production of SCFAs. FMT was also found to inhibit the growth of harmful bacteria and suppress the activation of the TGF-β1/Smad/ERK signaling pathway. However, there is a lack of clinical studies investigating the effects of FMT on renal function, highlighting the need for further research in this area [103]. Adverse events associated with FMT are linked to donor selection and transplantation methods. The timing and composition of FMT are subjects of ongoing debate [104]. The authors suggest that personalized FMT based on precise analysis of the microbiota of both the donor and recipient should be pursued, moving toward targeted transplantation treatments using specific gut microbiota [105].

#### 3.4.2. Expanded microbiota-based interventions in AKI

While current therapeutic interventions in AKI predominantly focus on probiotics and FMT, emerging microbiota-based strategies offer promising alternatives with potentially greater specificity and efficacy. These include engineered bacteria, prebiotics, and targeted modulation of microbial metabolites, which represent a significant advancement in the translational application of microbiome science.

*Engineered bacteria* are genetically modified microorganisms designed to perform targeted therapeutic actions. For example, *Escherichia coli* Nissle 1917 has been engineered to secrete anti-inflammatory cytokines such as IL-10, showing beneficial effects in preclinical models of intestinal and systemic inflammation. In the context of AKI, similar strategies may be employed to reduce renal inflammation or degrade uremic toxins. Notably, *Bifidobacterium longum* strains have been engineered to express urease, facilitating ammonia detoxification and mitigating hyperammonemia – conditions commonly exacerbated in AKI and advanced kidney failure [32,106,107].

*Prebiotics*, such as inulin, oligofructose, and resistant starch, selectively stimulate the growth of beneficial gut bacteria, including SCFA-producing species like *Bifidobacterium* and *Faecalibacterium*. In CKD patients, prebiotic supplementation has been shown to reduce serum levels of uremic toxins (e.g., IS, p-CS) while promoting SCFA production, which plays a crucial role in maintaining gut epithelial integrity and systemic immune modulation. In AKI models, prebiotic administration restored gut barrier function and reduced renal inflammation through GPR43-mediated signaling pathways [23,108].

Another innovative approach involves the *targeted modulation of microbial metabolites*. Metabolites such as SCFAs (butyrate, acetate, propionate), tryptophan-derived indoles, and TMAO have direct immunomodulatory and nephrotoxic implications. Strategies to enhance beneficial metabolite production or inhibit harmful ones are gaining traction. For instance, the compound 3,3-dimethyl-1-butanol (DMB) inhibits microbial TMA production, thus lowering TMAO levels and reducing fibrosis in kidney injury models. Similarly, butyrate analogs like tributyrin have demonstrated efficacy in attenuating ischemia–reperfusion-induced tubular injury by modulating histone acetylation and reducing pro-inflammatory gene expression [37,109].

Integrative strategies that combine probiotics, prebiotics, and metabolite-targeting agents offer synergistic potential. For example, co-administration of *Akkermansia muciniphila* with mucin-like prebiotics has been shown to enhance gut barrier function and reduce endotoxemia in septic AKI models. These advanced approaches highlight the translational potential of microbiome modulation in improving AKI outcomes beyond traditional interventions [48,110].

#### 3.4.3. Expanded molecular insights: intracellular signaling and metabolite transport

Specific intracellular signaling pathways and trans-epithelial transport mechanisms play crucial roles in mediating the interaction between gut-derived microbial metabolites and renal cell function. Understanding these mechanisms is critical for elucidating how gut dysbiosis exacerbates AKI and for developing targeted interventions. Among the most studied signaling cascades are the PI3K/AKT (phosphatidylinositol 3-kinase)/(protein kinase B) and MAPK (mitogen-activated protein kinase) pathways. Microbial metabolites, especially SCFAs, can modulate these pathways in renal tubular epithelial cells. SCFAs such as butyrate and acetate activate PI3K/AKT signaling, promoting anti-apoptotic responses, enhancing cell survival, and reducing oxidative stress in ischemic AKI models [111–113]. Conversely, microbial toxins like IS activate MAPK pathways, including p38 and ERK1/2, which are linked to pro-inflammatory cytokine production, oxidative stress, and tubular epithelial cell apoptosis [112,114]. These dichotomous effects highlight the importance of metabolite specificity and concentration in influencing renal outcomes. Transport of microbial metabolites across renal epithelial barriers also involves defined molecular systems. Notably, uremic toxins such as IS and p-CS are actively transported into renal cells via organic anion transporters (OAT1 and OAT3), which are predominantly expressed in proximal tubular cells [111,115]. These transporters facilitate intracellular toxin accumulation, leading to activation of TLR4–NF-κB signaling and increased pro-inflammatory cytokine release. Furthermore, the expression and activity of OATs may be downregulated during dysbiosis or AKI, which worsens toxin retention and renal injury [116]. By modulating these intracellular and epithelial mechanisms, gut microbiota-derived molecules directly influence renal pathology beyond systemic immune effects. Targeting these pathways through microbial or pharmacologic intervention represents a promising direction for AKI therapeutics.

#### 3.4.4. Multi-omics integration in AKI-microbiota research

The integration of multi-omics approaches – particularly metagenomics (profiling microbial taxonomy/functional potential via KEGG pathways, i.e., Kyoto Encyclopedia of Genes and Genomes) coupled with metabolomics (quantifying metabolites like SCFAs, TMAO, and uremic toxins) – has emerged as a transformative paradigm for decoding microbiome–host crosstalk in AKI. These methodologies synergistically link microbial taxonomic and functional shifts (metagenomics) to biochemical activity (metabolomics), offering unprecedented mechanistic insights into AKI pathogenesis [20]. For example, Andrianova et al. combined 16S rRNA sequencing with LC–MS metabolomics in ischemic AKI rats, revealing that *Staphylococcus*-enriched dysbiosis drives renal damage through accumulation of acylcarnitines [26]. Liu et al. integrated whole-genome sequencing and serum metabolomics to demonstrate *Akkermansia*-mediated bile acid dysregulation as a key mediator of post-AKI fibrosis [96]. In human septic AKI, correlated *Enterococcus* dominance (metagenomics) with elevated phenylacetylglutamine (metabolomics), directly linking pathobiont expansion to neutrophil hyperactivation. Critically, integrated signatures (e.g., ↓ Roseburia + ↓ butyrate + ↑ TMAO) show promise as early AKI biomarkers, outperforming traditional metrics like creatinine in preclinical models. However, human applications remain limited by technical barriers: longitudinal sampling in critically ill patients is challenging, and antibiotic confounders complicate metabolite attribution [117–119]. Future studies must prioritize standardized multi-omics frameworks – incorporating metatranscriptomics and proteomics – to resolve dynamic microbiota–renal cell interactions across AKI trajectories. Combined metagenomic–metabolomic phenotyping should be embedded in AKI cohort studies to identify actionable dysbiosis signatures – particularly in high-risk subgroups (sepsis, post-cardiac surgery). Multi-omics integration is indispensable for decoding microbiome-driven AKI pathogenesis. By bridging microbial genes to host metabolites, this approach uncovers actionable biomarkers and targeted therapies, advancing precision nephrology. Such advances could unlock personalized risk stratification and microbiome-targeted therapies.

#### 3.4.5. Barriers to human AKI microbiota studies

While mechanistic insights derive largely from animal models (e.g., GF mice), emerging clinical data confirm dysbiosis as a biomarker and therapeutic target in human AKI. We contextualize scarcity by addressing methodological and ethical challenges:

*Timing*: AKI is often acute/critical, limiting longitudinal sampling.

*Confounders*: Antibiotics, fluid resuscitation, and comorbidities alter microbiota independently.

*Ethics*: Invasive sampling (e.g., serial biopsies) is rarely justifiable in unstable patients. These constraints necessitate cautious extrapolation from models but do not invalidate preclinical findings – rather, they highlight the urgency of targeted human studies.

*Future directions to address scarcity*: We outline solutions to accelerate human research:

*Noninvasive sampling*: Fecal/plasma metabolomics (e.g., TMAO, SCFAs) as dynamic biomarkers.

*Multicenter cohorts*: Track microbiota shifts in AKI subphenotypes (septic vs. ischemic).

*Interventional trials*: Test engineered probiotics (e.g., butyrate-producing *E. coli*) in early-phase studies.

While animal models remain essential for mechanistic insights, our review integrates all available human evidence and identifies pathways for clinical translation. We agree that large-scale human studies are needed – a gap we explicitly frame as a research priority. By synthesizing preclinical rigor with emerging clinical data, this review bridges bench-to-bedside gaps in AKI-microbiota research.

## 4. Conclusions and future perspectives

Though little study has been done, there is growing evidence that the gut microbiota may have an impact on kidney function. Applying the latest discoveries in microbiome science to patients suffering from AKI may improve their prognoses. Personalized therapy strategies based on metabolomics and microbiota makeup might be possible. Prebiotics, probiotics, and synbiotics are examples of ‘natural’ medicines that aim to modulate the gut microbiota and may be useful in treating dysbiosis and getting rid of toxic metabolites. Furthermore, therapies aimed at the microbiome may lessen the risk of serious infections during AKI and AKI associated with sepsis. Combined metagenomic–metabolomic signatures show promise as AKI biomarkers, proposing that future studies prioritize standardized multi-omics frameworks.

In summary, research on the role of the gut microbiota in renal physiology and pathology has grown significantly in the last several years. Although there is evidence linking changes in the gut microbiota to disease, it is still unclear whether the microbiota is essentially bystanders in disease progression or whether we may purposefully manipulate them. To fully explore and reveal this burgeoning field’s promising potential, more research will be necessary.

*Limitations*: While there is increasing evidence on the significance of the microbiome in kidney illnesses, mechanistic and clinical data, especially those related to AKI, are still few.

To assess how probiotics and synbiotics affect the mortality rates of postoperative and critically ill patients, large-scale, rigorous randomized controlled studies are necessary. Because the extent and scope of the current trials are constrained, sufficiently powered research must be conducted. These kinds of trials are the only way we can get the data we needed to decide whether or not surgical and critically ill patients should have probiotics or synbiotics regularly.
